# A Multi-Objective Optimization Cutting Method for Irregularly Shaped Fish Fillets

**DOI:** 10.3390/foods15142423

**Published:** 2026-07-08

**Authors:** Xu Zhang, Yuanshan Zhao, Xinming Ma, Wenkai Wang, Zhuoyi Zhao, Guoxu Wang, Huihui Wang

**Affiliations:** 1 School of Mechanical Engineering & Automation, Dalian Polytechnic University, Dalian 116034, China; zhangxu_dlut@163.com (X.Z.); zhaoyuanshan2024@163.com (Y.Z.); xmm202706@163.com (X.M.); 18317736585@163.com (W.W.); zzy1234560421@163.com (Z.Z.); 17640531970@163.com (G.W.); 2 State Key Laboratory of Marine Food Processing and Safety Control, Dalian Polytechnic University, Dalian 116034, China

**Keywords:** irregular fish fillets, line-laser scanning, weight–length distribution model, multi-objective cutting optimization, intelligent fish processing

## Abstract

Irregularly shaped fish fillets make it difficult for conventional cutting methods to simultaneously control slice weight, maintain shape consistency, and reduce residual material. To address this problem, this study proposed a multi-objective optimization cutting method based on line-laser scanning and mathematical modeling. First, three-dimensional point-cloud data of fish fillets were acquired, and a weight–length distribution model was established to describe the mass distribution along the fillet length. Then, a constrained multi-objective cutting model was developed by taking slice weight as a constraint and minimizing both the variation in slice cross-sectional area and the residual material weight. An improved Marine Predators Algorithm combined with Deb’s feasibility rules was used to solve the cutting-position optimization problem. The results showed that the weight prediction accuracy of the developed distribution model reached 94.4–96.6%. Compared with the conventional sequential cutting method, the proposed method reduced the relative standard deviation of slice cross-sectional area to 8.900%, 11.112%, and 13.601% at target slice weights of 10 g, 15 g, and 20 g, respectively. Meanwhile, the average residual material weights were reduced to 0.581, 0.693, and 0.710 times the theoretical residual quantities, and the mean relative errors of slice weight were all below 8%. These findings indicate that the proposed method can improve slice shape consistency and raw material utilization while maintaining acceptable weight accuracy, providing a useful approach for intelligent and standardized fish fillet cutting.

## 1. Introduction

Fillet cutting is a key operation in the pretreatment of fish raw materials, aiming to obtain slices or portions with specific requirements in terms of weight, shape, and other specifications for use in ready-to-eat products or as raw materials for marinating, smoking, drying, and thermal processing [1]. As high-quality and standardized deep-processed fish products have become increasingly favored by consumers, precise cutting of fish fillets has assumed an important role in improving both product quality and economic value [2]. Accurate control of slice weight is beneficial for improving the quality of seasoning penetration and thermal processing, while control of slice shape consistency enhances product appearance and increases added value. In addition, reducing residual material weight generated during fillet cutting is of great importance for improving processing efficiency and economic performance [3]. It is well known that, after filleting, the overall shape of fish fillets is typically wide and thick in the middle, particularly in the abdominal region, and gradually becomes narrower and thinner toward both ends, especially toward the tail [4]. Therefore, fish fillets are not only irregularly shaped but also subject to individual variation, which creates substantial technical challenges for precise cutting under different processing requirements [5]. Owing to the lack of real-time detection of fillet shape, conventional fish fillet cutting equipment can only perform simple cutting at fixed intervals and cannot achieve precise cutting [6]. In actual production, complex fish fillet cutting still relies heavily on manual operation. This process is labor-intensive and time-consuming, and it cannot ensure cutting accuracy or product consistency. With the development of advanced measurement technologies, line-laser scanning has been introduced for fillet shape measurement [7]. By reconstructing real-time geometric data of complex fillet shapes and establishing data-driven models of weight distribution, fixed-weight vertical cutting of fish fillets has become possible, greatly improving the intelligence level of cutting equipment. However, such approaches still cannot simultaneously satisfy diversified requirements related to slice weight, shape consistency, and residual material weight. Therefore, further improvement and innovation in fish fillet cutting technology are highly necessary [8]. A primary technical challenge is how to effectively utilize fillet shape information to achieve optimized cutting that balances multiple objectives through the coordinated control of parameters such as slice thickness and cutting angle. Addressing this challenge can provide a technical basis for the development of intelligent cutting equipment for fish raw materials.

Existing studies on the precise cutting of irregularly shaped fish fillets have mainly focused on the acquisition of geometric parameters of raw materials, modeling of the relationship between weight and geometric features, and optimization of cutting planning [9]. In the area of shape measurement and weight prediction, Tarcila et al. developed a multiple linear regression model based on morphological features of salmon, including body length, body height, and body width, to predict fish weight. However, such methods rely heavily on manual feature extraction, and their robustness and online applicability are limited when individual variation among raw materials is large [10]. Bianca et al. employed machine vision combined with image segmentation and a random forest algorithm to estimate cod weight, demonstrating the feasibility of using visual information for fish weight prediction. Nevertheless, two-dimensional images provide limited representation of geometric information for complex curved surfaces and show insufficient stability under dynamic conveying conditions and fluctuating illumination [11]. To improve the accuracy of geometric parameter acquisition for fish raw materials with complex curved surfaces, Shi et al. further conducted studies on fish surface contour reconstruction using line-laser scanning and enhanced edge contour clarity through point-cloud optimization, thereby providing technical support for the non-contact acquisition of three-dimensional shape information from irregular fish fillets [12]. Recent studies have also attempted to determine fish cutting positions using visual or three-dimensional geometric information. For example, Jang and Seo predicted fish cutting points according to target weight using machine vision [13], while Zhang et al. used line-laser scanning combined with machine learning to identify fish head cutting positions [14]. In terms of cutting planning, Chen et al. introduced a genetic algorithm into the dual-objective optimization of fish cutting in terms of weight and size, achieving coordinated control of slice weight and size. However, this modeling process was based on idealized assumptions, such as uniform fish density, and thus could not accurately characterize the actual features of fish fillets, which exhibit substantial variations in width and thickness along the length direction and non-uniform weight distribution [15]. Overall, although existing studies have provided technical support for precise fish cutting from measurement to modeling and optimization, there is still a lack of a systematic solution that effectively integrates three-dimensional shape measurement, weight distribution modeling, and constrained multi-objective optimization [16]. In particular, the intrinsic relationship between cutting control variables and the processing objectives related to quality and economic performance has not yet been fully clarified, and effective cutting planning methods for the high-quality processing of irregularly shaped fish fillets remain inadequate [17].

To address these issues, this study aimed to develop a multi-objective optimization cutting method for fish fillets that improves both processing quality and economic performance. Specifically, a line-laser scanning system was used to acquire surface point-cloud data and reconstruct fillet contours. A weight–length distribution model was then developed to describe the geometric and mass distribution characteristics of irregular fish fillets. Based on this model, a constrained multi-objective cutting model was constructed to minimize slice cross-sectional area variation and residual material while maintaining slice weight within the required range. The model was solved using an MPA combined with Deb’s feasibility rules and was validated against the conventional sequential cutting method. Compared with previous studies that mainly addressed geometric measurement, weight prediction, or cutting-position determination separately, this study integrates three-dimensional line-laser measurement, weight–length distribution modeling, and constrained multi-objective cutting optimization into a unified framework. By treating slice weight as a constraint and using cutting interval and cutting angle as decision variables, the proposed method aims to improve slice shape consistency and raw material utilization while maintaining acceptable weight accuracy.

## 2. Materials and Methods

### 2.1. Sample Preparation

The experimental material consisted of mackerel fillets obtained after filleting, which were purchased from the Jinsanjiao Aquatic Products Market in Dalian, China. A total of 40 chilled mackerel with intact appearance were randomly selected, with an average body weight of 768.0 ± 54.2 g and an average body length of 33.00 ± 2.32 cm. The fish were filleted, and the flesh on both sides of the backbone was separated, yielding a total of 80 fillet samples for the experiments. Mackerel fillets were selected because they are common economic fish materials in industrial processing and show typical irregular geometry after filleting. The fillets usually have a relatively wide and thick abdominal region and a gradually narrowing tail region, which makes it difficult for cutting methods based only on slice length or target weight to simultaneously achieve weight accuracy, shape consistency, and residual material reduction. Therefore, mackerel fillets provide a suitable material for evaluating the proposed multi-objective cutting framework. Since the proposed method is based on fillet shape acquisition and weight–length distribution modeling, it may be extended to other fish fillets with similar geometric characteristics after corresponding density measurement, model fitting, and cutting-parameter validation. Among them, 10 fillets were used for density measurement, 10 fillets were used for validation of the volume and weight distribution functions, and the remaining 60 fillets were equally divided into three groups for cutting validation experiments with target weights of 10 g, 15 g, and 20 g. The sample allocation was designed according to the experimental purpose of each stage. The 10 fillets obtained from the same species, source, and chilled storage condition were used to determine the average density of the fillets. Another 10 fillets were cut at 10 mm intervals, providing more than 250 local volume/weight measurements along the fillet length for evaluating the accuracy of the volume/weight–length distribution models. The 60 fillets used for cutting validation were equally divided into three target-weight groups of 10 g, 15 g, and 20 g, and the samples in each group were cut according to the cutting schemes generated by the proposed method and the Classic Sequential Algorithm, respectively. Therefore, the current sample size can support the methodological validation and comparative evaluation under the tested conditions. From the perspective of framework-level transferability, generalized application of the proposed method in fish cutting still requires density measurement, distribution-model fitting, and cutting-parameter validation for different fish species, sources, fishing seasons, or temperature conditions.

### 2.2. Data Acquisition

**Figure 1:**
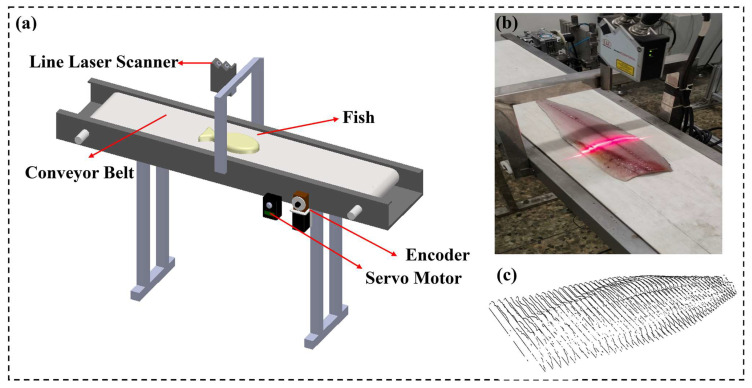
Data acquisition process: (**a**) line-laser scanning system; (**b**) data acquisition setup; (**c**) acquired point-cloud data.

In this study, a line-laser scanning system was employed to acquire three-dimensional contour information of the fish fillets. As shown in Figure 1a, the system mainly consisted of a conveying mechanism, a line-laser scanner, and an encoder. The conveying mechanism comprised a conveyor belt driven by a servo motor, which enabled the horizontal transport of the fillet samples. The conveyor speed was set to 0.05 m/s based on preliminary equipment commissioning and scanning tests. At this speed, the fillets moved smoothly through the scanning area, and the acquired point clouds were sufficiently dense for contour reconstruction and weight–length distribution modeling. Under the fixed sampling frequency used in this study, increasing the conveyor speed would enlarge the spacing between adjacent scanned profiles and may reduce the description of local contour details, whereas a lower speed would increase data redundancy and acquisition time. Therefore, the conveyor speed was fixed at 0.05 m/s in all experiments to keep the data acquisition conditions consistent [18]. A support frame was installed at the midpoint of the conveyor belt, on which the line-laser scanner was mounted. After calibration, the scanner projected a line laser vertically downward. The line-laser scanner used in this study was an LLT-2600 SCANCONTROL 3D scanner (Micro-Epsilon Messtechnik GmbH & Co. KG, Ortenburg, Germany) equipped with a 658 nm red line laser. Before data acquisition, the scanner and conveyor system were calibrated using a standard reference plate. After calibration, the effective spatial resolution of the acquired point cloud was approximately 0.1 mm in the profile direction and 0.5 mm along the conveying direction, and the calibration error was controlled within 0.2 mm. Depending on the fillet size, each reconstructed fillet point cloud contained approximately 3.0 × 10^4^ to 5.0 × 10^4^ points after filtering and resampling. Point-cloud preprocessing, contour reconstruction, volume calculation, and weight–length distribution modeling were performed using self-developed Python programs developed and executed in Python 3.9.21 and PyCharm 2024.2.1 Community Edition. The encoder was used to record the conveyor displacement in real time and to assist in the spatial registration of the point-cloud data [19]. To balance contour reconstruction accuracy and data-processing efficiency, the sampling frequency of the line-laser scanning system was set to 0.4 kHz. At a conveyor speed of 0.05 m/s, this sampling frequency corresponds to an interval of approximately 0.125 mm between adjacent scanned profiles along the conveying direction. This profile spacing was much smaller than the main geometric variation scale of the fillets and was sufficient to capture the surface contour changes required for volume reconstruction and weight–length distribution modeling. Preliminary scanning tests showed no obvious contour discontinuity or loss of major geometric features. Although a higher sampling frequency could further increase point-cloud density, it would also increase data volume and processing cost. Therefore, 0.4 kHz was used as a practical compromise between contour reconstruction accuracy and data-processing efficiency [20]. As shown in Figure 1b, the fillet sample was placed on the conveyor belt and scanned after the line-laser scanner was triggered, thereby obtaining the surface contour point-cloud data shown in Figure 1c [21].

### 2.3. Weight Distribution Model of Fish Fillets

The mathematical relationship between the weight distribution of fish fillets and their geometric shape is the basis for constructing the mathematical model for the multi-objective optimization cutting problem. Fish fillets are irregular biological materials and exhibit individual variability. However, their weight distribution is still related to geometric characteristics and can therefore be described quantitatively using a mathematical function.

Based on the surface contour point-cloud data of fish fillets acquired in Section 2.2, a distribution model describing the variation in fillet weight along the length direction was established according to the principle of calculus. Let the length of the fillet along the conveyor direction be L [22]. The fillet was divided vertically into N slices with equal thickness and small dimensions, and the slice thickness Δh can be expressed as Equation (1).

(1) $$\Delta \;h=\frac{L}{N}\;\;$$

The volume of each slice was calculated using the water-filling method, in which the slice was virtually filled with water, and the slice volume was obtained from the volume of water required to completely fill it [23]. The position $l_{i}$ and volume change rate $\Delta v_{i}$ of each slice can be expressed as Equations (2) and (3), respectively.

(2) $$l_{i}=\sum_{i=1}^{N}\Delta h$$

(3) $$v_{i}=\frac{∆v_{i}}{∆h}\;\;i=1,\ldots ,N$$

A least-squares polynomial fitting was then performed on the volume change rates and positional data of the N slices to obtain the volume–length distribution function of the fish fillet, as shown in Equation (4). In this equation, $a_{0}$, $a_{1}$, $a_{2}$, …, $a_{n}$ are polynomial coefficients, and a fifth-order polynomial was adopted in this study. Preliminary fitting tests showed that lower-order polynomials could not fully describe the curvature variation in the abdominal region, whereas higher-order polynomials tended to produce local oscillations near the head and tail. Therefore, the fifth-order polynomial was selected to balance fitting accuracy and model stability, and possible overfitting was checked by comparing the fitted curves with the measured segment data and residual distribution.

*v*(*l*) = *a*_0_ + *a*_1_*l* + *a*_2_*l*^2^ + ⋯ + *a_n_l^n^*,    0 ≤ *l* ≤ *L* (4)

By treating the fish fillet as a body with uniform density, the fillet weight was measured using an electronic balance and the fillet volume was measured by the water displacement method, from which the density ρ of the fillet was calculated [24]. Substituting ρ into the volume–length distribution function yielded the weight–length distribution function w(l) of the fish fillet, as given in Equation (5). In this study, the average density of the whole fillet was used for the conversion from volume distribution to weight distribution. This treatment was adopted mainly to improve the engineering applicability of the proposed method. Differences in moisture, fat, and other tissue components among different fillet regions may lead to non-uniform density within individual fillets. In addition, the density of fish fillets from different batches may vary with species, source, fishing season, and storage condition. Given these factors, it is difficult to establish a generalized density-distribution model that is applicable across different fish species and processing conditions. Therefore, for fish raw materials from the same source and storage condition, the use of average density obtained by conventional density measurement provides a practical way to establish a targeted weight–length distribution model.

(5) $$w\left(l\right)=\rho v\left(l\right)$$

### 2.4. Mathematical Model of the Multi-Objective Fish Fillet Cutting Problem

The technical requirements for precise fish fillet cutting vary across application scenarios. However, the common goals are to improve processing quality and economic performance by enhancing slice weight and shape consistency and reducing residual material. With these objectives, a mathematical model for multi-objective optimization cutting was established to transform the cutting of irregularly shaped fish fillets into a solvable mathematical problem, thereby achieving coordinated optimization of slice weight, shape consistency, and residual material weight. In the model construction process, decision variables were first defined, followed by the formulation of objective functions and constraints. The resulting constrained optimization model was solved using an improved Marine Predators Algorithm (MPA) incorporating Deb’s feasibility rules [25].

#### 2.4.1. Decision Variables and Parameter Calculation

**Figure 2:**
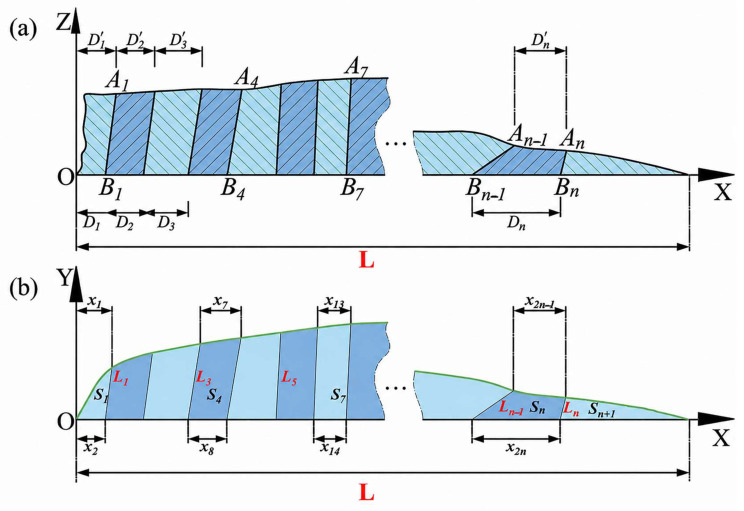
Simplified schematic illustration of the cutting-scheme definition: (**a**) representative cutting sections and cutting positions in the geometric projection; (**b**) representative cutting variables and their relationship with the weight–length distribution function.

For irregularly shaped fish raw materials, optimization of the cutting scheme relies on the effective establishment of the mathematical relationship between cutting objectives and cutting variables. Previous studies have shown that shape information obtained by laser scanning or machine vision, together with practical cutting requirements, provides an important basis for reasonably defining decision variables and achieving precise cutting of complex-shaped food materials [26,27]. Therefore, in this study, the cutting interval and cutting angle were selected as the main control variables for the cutting scheme. They affect slice weight, slice geometry, and residual material weight. A specific cutting scheme can be obtained by assigning values to these control variables. Figure 2 provides a simplified schematic of the cutting-scheme definition, showing the representative cutting sections, cutting positions, and their relationship with the weight–length distribution function. For readability, repeated labels are omitted, and only representative cutting sections and variables are retained in the figure. As shown in Figure 2a, the projection of the cutting scheme in the X-Z plane is used to describe the geometric relationship of the cuts, where the *X*-axis represents the length direction and the *Z*-axis represents the height direction. A fish fillet with a length of L is cut n times and divided into n+1 parts, including n slices and one residual material portion, and the cutting sections are denoted sequentially as $A_{1}B_{1},A_{2}B_{2},\ldots ,A_{n}B_{n}$. The decision variables $D_{i}^{\prime }$ and $D_{i}$ are defined as the displacements of the starting and ending positions of the current cut relative to those of the previous cut, respectively. Evidently, different values of the control variables correspond uniquely to different starting and ending positions of the cuts. Because direct optimization of the control variables leads to a relatively complex modeling process, the control variables are transformed into the decision variables $D_{i}^{\prime }$ and $D_{i}$ to simplify the calculation. According to the above analysis of diversified cutting requirements for fish fillets, the decision parameters were determined as slice weight, slice cross-sectional area, and residual material weight. To clarify the relationship between the decision parameters and the variables, the weight–length distribution function w(l) established in Section 2.3 was introduced, as shown in Figure 2b. Taking the foremost end of the fillet as the origin, the X-axis denotes the length direction, and the Y-axis denotes the rate of weight variation. The cutting variables $D_{i}^{\prime }$ and $D_{i}$ are transformed into the decision variables $x_{2i-1}$ and $x_{2i}$, and the cutting sections in the cutting scheme correspond sequentially to the cutting lines $L_{1},L_{2},\ldots ,L_{n}$ in Figure 2b. According to the principle of calculus, the areas $S_{1},S_{2},\ldots ,S_{n}$, and $S_{n+1}$, enclosed by the weight–length distribution function and the cutting lines in Figure 2b, represent the weights of the slices and the residual material portion [28]. Therefore, the decision variable vector to be solved in this study is $x=[D_{1},D_{1}^{\prime },D_{2},D_{2}^{\prime },\ldots ,D_{n},D_{n}^{\prime }]=[x_{1},x_{2},x_{3},\ldots ,x_{2n}]$.

**Figure 3:**
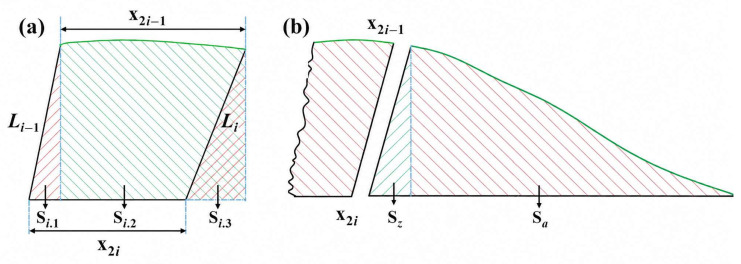
Local schematic illustration for weight calculation: (**a**) enclosed region corresponding to one slice; (**b**) enclosed region corresponding to the residual material portion.

(1) Slice Weight:

Figure 3a shows the weight–length distribution function curve for the i-th slice. According to the principle of calculus, the weight $W_{i}$ of the i-th slice is equal to the area $S_{i}$ enclosed by the corresponding segment of the weight–length distribution function and the cutting lines $L_{i-1}$ and $L_{i}$. The calculation formula is given in Equation (6).

(6) $$W_{i}=S_{i}=\left\{\begin{array}{c} \begin{array}{cc} S_{i2}+S_{i3} & i=1 \end{array}\\ \begin{array}{cc} S_{i1}+S_{i2}-S_{i3}, & i=2,3,\cdots ,n \end{array} \end{array}\right.$$

In Equation (6), $S_{i2}$ denotes the area under the weight distribution function curve corresponding to the i-th slice, while $S_{i1}$ and $S_{i3}$ denote the areas under the cutting lines $L_{i-1}$ and $L_{i}$, respectively. $S_{i1}$, $S_{i2}$, and $S_{i3}$ can be expressed by Equations (7)–(9), respectively.

(7) $$\begin{array}{cc} S_{i2}=\int_{\sum_{j=1}^{i-1}x_{2j-1}}^{\sum_{j=1}^{i}x_{2j-1}}w\left(x\right), & i=1,2,\cdots ,n \end{array}$$

(8) $$\begin{array}{cc} S_{i1}=\frac{1}{2}\left(\sum_{j=1}^{i-1}x_{2j-1}-\sum_{1}^{i-1}x_{2j}\right)w\left(\sum_{j=1}^{i-1}x_{2j-1}\right), & i=2,3,\cdots ,n \end{array}$$

(9) $$\begin{array}{cc} S_{i3}=\frac{1}{2}\left(\sum_{j=1}^{i}x_{2j-1}-\sum_{1}^{i}x_{2j}\right)w\left(\sum_{j=1}^{i}x_{2j-1}\right), & i=1,2,\cdots ,n \end{array}$$

Here, x_2j−1_ and x_2j_ represent the cutting intervals of the j-th slice at the initial and final cutting positions, respectively, and w(x) denotes the rate of weight variation at the fillet length position x. As illustrated in Figure 3a, $S_{i2}$ represents the basic volume obtained under vertical cutting. For inclined cutting, the actual slice volume $S_{i}$ is corrected based on $S_{i2}$. Specifically, the volume below the previous cutting section, $S_{i1}$, is added, while the volume below the next cutting section, $S_{i3}$, is subtracted. Therefore, the inclined-cut slice volume can be regarded as the corrected result obtained from the vertical-cut volume.

(2) Slice Cross-Sectional Area:

As shown in Figure 3a, the lengths of the cutting lines $L_{i-1}$ and $L_{i}$ represent the areas of the two cutting surfaces of the i-th slice. Because the cutting angle may vary, the areas of the two cutting surfaces of the same slice may differ. Therefore, their average value was used. The average cross-sectional area of the i-th slice can be expressed by Equation (10).

(10) $$L_{i}^{\prime }=\left\{\begin{array}{c} \begin{array}{cc} L_{1} & i=1 \end{array}\\ \begin{array}{cc} \frac{L_{i-1}+L_{i}}{2}, & i=2,3,\cdots ,n \end{array} \end{array}\right.$$

In Equation (10), $L_{i}$ denotes the cross-sectional area corresponding to the i-th cut, which can be expressed by Equation (11).

(11) $$\begin{array}{cc} L_{i}=\sqrt{{\left(\sum_{1}^{i}x_{2j-1}-\sum_{1}^{i}x_{2j}\right)}^{2}+{\left(w\left(\sum_{1}^{i}x_{2j-1}\right)\right)}^{2}}, & i=1,2,\cdots ,n \end{array}$$

(3) Residual Material Weight:

When fish fillets are cut sequentially toward the tail, the fillets gradually become narrower and thinner. Although cutting can still be continued by increasing the cutting length and the inclination angle of the cut, the resulting portions may be discarded because of poor appearance quality and reduced consistency with the other slices, or because the remaining fillet is too small to form one complete slice. Such discarded material is referred to as residual material. Residual material weight is an important indicator for evaluating the economic performance of the cutting process. As shown in Figure 3b, the residual material weight SB can be expressed as the area SB enclosed by the cutting line segment of the n-th cut and the weight distribution function curve of the remaining fillet. The residual material weight can be expressed by Equation (12).

(12) $$W_{B}=S_{a}+S_{z}$$

In Equation (12), $S_{a}$ denotes the area under the weight distribution function curve of the residual material portion, and $S_{z}$ denotes the area under the cutting line $L_{n}$. The expressions for $S_{a}$ and $S_{z}$ are given in Equations (13) and (14), respectively.

(13) $$S_{a}=\int_{\sum_{j=1}^{n}x_{2j-1}}^{L}w\left(x\right)$$

(14) $$S_{z}=\frac{1}{2}\left(\sum_{j=1}^{n}x_{2j-1}-\sum_{j=1}^{n}x_{2j}\right)w\left(\sum_{j=1}^{n}x_{2j-1}\right)$$

#### 2.4.2. Objective Functions and Constraint Conditions

In multi-objective optimization problems, the constraint optimization method can be used to convert one of the objectives into a constraint condition, thereby facilitating the solution process. Among the three decision parameters in the multi-objective fish fillet cutting problem, slice weight must be controlled within a reasonable range. Therefore, slice weight was treated as a constraint, whereas slice cross-sectional area and residual material quantity were defined as the optimization objectives. During fish fillet cutting, the greater the similarity in slice area, that is, the smaller the dispersion of slice area values, the better the consistency of slice shape specifications [29]. Therefore, the relative standard deviation of slice area within the same batch was introduced to characterize the dispersion of slice cross-sectional area after cutting. In this study, “shape consistency” refers to geometric specification consistency rather than sensory or consumer-oriented appearance quality. Although aspect ratio and curvature uniformity can describe visual appearance, they are sensitive to natural contour variation in irregular fillets and are less suitable for consistent comparison among samples. Under the weight constraint used in this study, cross-sectional area is more directly related to slice size and thickness uniformity; therefore, the relative standard deviation of slice cross-sectional area was used to evaluate the batch-level dispersion of slice specifications. The objective function for slice cross-sectional area, $f_{1}(x)$, can be expressed as Equation (15).

(15) $$f_{1}(x)=\frac{\sigma }{{\bar{L}}^{\prime }}$$

In Equation (15), σ and ${\bar{L}}^{\prime }$ denote the standard deviation and mean value of slice cross-sectional area within the same batch, respectively, and their expressions are given in Equations (16) and (17) [30].

(16) $$\sigma =\sqrt{\frac{\sum_{i=1}^{n}\left(L_{i}^{\prime }-{\bar{L}}^{\prime }\right)}{n-1}}$$

(17) $${\bar{L}}^{\prime }=\frac{L^{\prime }}{n}$$

When cutting fish fillets, smaller residual material weights are preferred. To unify the dimensions of the objective functions, the residual material weight function was normalized, and the optimization objective for residual material weight was defined as the relative residual material weight, i.e., the ratio of residual material weight to the total weight of the fillet [31]. The objective function for residual material weight, $f_{2}(x)$, can be expressed by Equation (18).

(18) $$f_{2}\left(x\right)=\frac{S_{B}}{\int_{o}^{L}w(x)}$$

A weighted-sum method was used to combine the two objectives: minimizing the relative standard deviation of slice cross-sectional area and minimizing the relative residual material quantity, as shown in Equation (19) [32].

(19) $$\begin{array}{c} \min\;f(x)=\omega_{1}f_{1}(x)+\omega_{2}f_{2}\left(x\right)\\ s.t.\;g_{i}(x)=\frac{\left|W_{i}-W_{a}\right|}{W_{a}}-0.05\leq 0\\ g_{n+1}\left(x\right)=\sum_{j=1}^{i}x_{2k}-L<0\\ g_{n+2}\left(x\right)=\sum_{j=1}^{i}x_{2k-1}-L<0\\ g_{n+2+i}(x)=-x_{2j}<0\\ g_{2n+2+i}\left(x\right)=-x_{2j-1}<0\\ g_{3n+2+i}\left(x\right)=\sum_{j=1}^{i}x_{2k}-\sum_{j=1}^{i}x_{2k-1}\leq 0\\ {g_{4n+3}\left(x\right)=S}_{B}-0.95S_{a}<0 \end{array}$$

**Figure 4:**
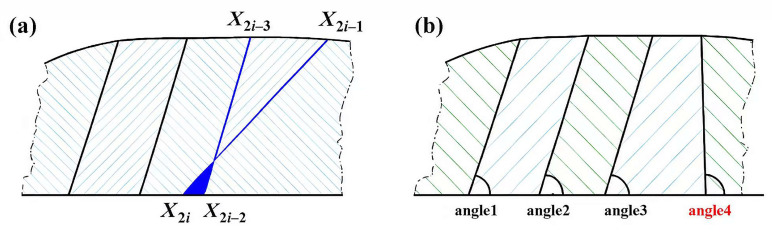
Examples of incorrect cutting configurations: (**a**) cutting interference; (**b**) an obtuse cutting angle.

Here, i=1,2,3,…,n. $\omega_{1}$ and $\omega_{2}$ are the weights of the objective functions. The weighting coefficients were determined through a sensitivity comparison of different coefficient combinations. Increasing the weight of the slice-area objective improved slice specification consistency, whereas increasing the weight of the residual-material objective further reduced residual material but increased the dispersion of slice cross-sectional area. Therefore, the weights of the slice-area objective and residual-material objective were set to 0.7 and 0.3, respectively, as a practical trade-off between slice specification consistency and raw material utilization. These coefficients can be adjusted according to different production priorities and product specifications. $g_{i}(x)$ denotes the slice weight constraint. To ensure high cutting accuracy, the slice weight error was restricted to within 5%. $g_{n+1}(x)$ and $g_{n+2}(x)$ constrain the search ranges of the cutting start and end positions so that they do not exceed the total length of the fillet. $g_{n+2+i}(x)$ and $g_{2n+2+i}(x)$ constrain the slice thicknesses at the cutting start and end positions to be greater than 0, thereby restricting the search for feasible solutions to the length range of the fillet and avoiding interference between adjacent cutting planes, as illustrated in Figure 4a. As shown in Figure 4b, when slice cross-sectional area is used as an optimization objective, cutting schemes with either acute or obtuse cutting angles may both be identified as feasible solutions. To improve slice shape consistency, the cutting angle must therefore be constrained, and $g_{3n+2+i}(x)$ is used to restrict the angle between the cutting plane and the fillet length direction to no more than 90°. $g_{4n+3}(x)$ constrains the residual material quantity to be less than the prescribed minimum slice weight, which serves as the termination condition for cutting and helps improve raw material utilization [33].

#### 2.4.3. Algorithm Implementation and Optimization

To solve the constrained multi-objective cutting model shown in Equation (19), an improved Marine Predators Algorithm (MPA) incorporating Deb’s feasibility rules was employed to optimize the cutting positions. MPA was selected because the cutting model contains continuous decision variables, nonlinear objective functions, and multiple constraints related to slice weight, residual material, cutting position, and cutting angle. The algorithm provides a balance between global exploration and local exploitation with relatively few control parameters, which is suitable for searching feasible cutting-position combinations [34]. Compared with GA and PSO, MPA requires fewer control parameters and has a clearer exploration–exploitation transition mechanism. Compared with NSGA-II, the present model does not aim to output a Pareto solution set for later manual selection; instead, slice weight is treated as a constraint, and the two remaining objectives are integrated into a weighted optimization model to generate one executable cutting scheme. Compared with simulated annealing, MPA uses a population-based search structure, which is more suitable for exploring multiple feasible cutting-position combinations [35]. Therefore, MPA combined with Deb’s feasibility rules was selected as the solver for the constrained cutting-position optimization problem in this study. In this algorithm, the starting and ending positions of each cut were taken as the search variables. Under the constraint conditions of the optimization model, coordinated optimization of slice specification variation and residual material weights was achieved. First, the initial population was randomly generated within the upper and lower bounds of each decision variable, and the population initialization scheme is given in Equation (20).

(20) $${Prey}_{0}=lb+rand(ub-lb)$$

In Equation (20), ${Prey}_{0}$ denotes the generated initial prey matrix, and ub and lb represent the upper and lower bounds of the search space, respectively; rand denotes a uniformly distributed random vector within the interval $\left[0,1\right]$. Subsequently, during the iterative process, the positions of candidate solutions were updated by simulating the dynamic interaction between predators and prey. The position updating strategy is given in Equations (21) and (22).

(21) $${step}_{i}=R_{b}⨂(Elite-R_{B}⨂{Prey}_{i})$$

(22) $${Prey}_{i}={Prey}_{i}+p\cdot R⨂{step}_{i}$$

In Equations (21) and (22), $Prey_{i}$ denotes the position vector of the i-th individual at the current iteration, $step_{i}$ denotes the movement step of the current prey, Elite denotes the current best position vector, $R_{B}$ denotes a random normal-distribution vector following Brownian motion, R denotes a random vector within the interval $\left[0,1\right]$, and P is a constant set to 0.5. Equation (21) is mainly used to enhance the global exploration ability of the population in the early stage of the search, whereas Equation (22) is used to improve the local exploitation ability in promising regions during the subsequent iterations, thereby achieving a balance between global exploration and local exploitation [36].

For each candidate cutting scheme, the slice weight, cross-sectional area, and residual material quantity were calculated according to Equations (6)–(18), and the resulting values were substituted into Equation (19) to obtain the overall objective function value, thereby completing the fitness evaluation of the candidate solution. Since the standard MPA is primarily designed for unconstrained continuous optimization problems, whereas the present model involves both slice weight and residual material quantity constraints as well as geometric constraints on cutting positions, Deb’s feasibility rules were introduced during candidate-solution comparison and population updating to screen feasible solutions. The constraint violation of a candidate solution is defined by Equation (23).

(23) $$f_{v}\left(x\right)=\sum_{i=1}^{4n+3}{\left({\langle x\rangle }_{i}\right)}^{{\langle x\rangle }_{i}^{o}}$$

In Equation (23), $\left\langle x\rangle_{i}=max\{0,g_{i}(x)\right\}$ denotes the violation amount of the i-th constraint, where i=1,2,3,…,4n+3. $\langle x\rangle_{i}^{o}$ denotes the order corresponding to the violation amount of the associated constraint. When $\langle x\rangle_{i}\leq 1$, $\langle x\rangle_{i}^{o}=1$; otherwise, $\langle x\rangle_{i}^{o}=2$. Here, $g_{i}(x)$ denotes the i-th constraint function, $f_{v}(x)$ denotes the constraint violation function of solution x, and 4n+3 is the total number of constraints. When x is a feasible solution, $f_{v}(x)=0$. When only one of two candidate solutions satisfies the constraints, the feasible solution is preferentially retained. When both candidate solutions satisfy the constraints, the one with the better objective function value is retained. When neither candidate solution satisfies the constraints, the one with the smaller constraint violation is retained [37]. Through this strategy, interference from infeasible solutions with the search direction can be effectively avoided, thereby improving the solution stability and optimization efficiency of the constrained cutting model. Once the termination criterion is met, the cutting-position combination corresponding to the best solution obtained during the iterative process is output as the final cutting scheme, from which the slice weight, cross-sectional area, and residual material quantity satisfying the constraints are determined. For reproducibility, the population size was set to 10, and the maximum number of iterations was set to 600. Each optimization case was independently repeated 10 times with different random initial populations. The search-space dimension was 2n, where n represents the theoretical number of slices for a given fillet. The algorithm was terminated when the maximum number of iterations was reached. The same population size, iteration number, and initialization strategy were used for both Standard MPA and Deb-MPA in the convergence comparison.

### 2.5. Evaluation Methods

#### 2.5.1. Experimental Validation of the Weight–Length Distribution Model

Ten fish fillet samples were selected for experimental validation. Each sample was scanned using the line-laser scanning system, and the predicted volume of each sample was obtained according to Equation (4). The actual weight and volume of the experimental samples were measured using an electronic balance and the water displacement method, respectively, and the average density was then calculated. The model-calculated volumes were compared with the measured values for analysis. The model performance was further evaluated using the squared correlation coefficient (R^2^), root mean square error (RMSE), mean absolute error (MAE), and residuals between the measured and model-calculated weights.

According to Equation (5), the weight–length distribution function of each sample was obtained by substituting the average density into the volume–length distribution function. The ten samples were then cut at equal intervals of 10 mm, and the volume and weight of each fish fillet segment were measured.

#### 2.5.2. Experimental Validation of the Multi-Objective Optimization Cutting Method

Existing intelligent fish fillet cutting equipment based on line-laser scanning technology is mainly used for fixed-weight cutting under vertical cutting conditions and is generally designed for single-objective requirements. Among these methods, the Classic Sequential Algorithm (CSA) is one of the most commonly used approaches [38]. The working principle of the CSA is as follows. Assuming that the fish fillet is vertically divided into small slices of equal thickness, the volume–length distribution function is first established according to the principle of calculus, as expressed in Equation (4), and the corresponding weight–length distribution function is then obtained according to Equation (5). The weight of each small slice is calculated using the weight–length distribution function and accumulated sequentially. During the accumulation process, the cumulative weight is compared with the prescribed target weight. When the absolute difference between the two values is smaller than the allowable error ε, the accumulation process for the current slice is terminated. The sum of the cumulative step lengths is then calculated to determine the horizontal movement distance required by the cutting tool for each cutting operation. This procedure is repeated until the remaining fillet weight is smaller than the prescribed target weight. The final output is the slice thickness sequence generated by the CSA.

The principal advantage of the Classic Sequential Algorithm is its accurate control of slice weight, whereas the multi-objective cutting method seeks to improve overall cutting performance through the coordinated optimization and trade-off of multiple decision parameters. The Classic Sequential Algorithm was selected as the reference cutting strategy because it represents a commonly used target-weight vertical cutting method in line-laser-based portioning systems and can be applied to the same fillet data and weight constraints. Therefore, it provides a direct baseline for evaluating whether the proposed method can improve slice shape consistency and reduce residual material while maintaining acceptable weight-control accuracy. To further examine the optimization solver, Deb-MPA was also compared with PSO and GA under the same objective function, constraints, population size, and maximum iteration number. Therefore, the comparison between the cutting schemes generated by the two methods was performed to evaluate whether the proposed method could improve the other two decision parameters and demonstrate general applicability while maintaining acceptable weight-control accuracy.

Specifically, 60 fish fillet samples were selected and scanned using the line-laser scanning system to obtain the weight–length distribution function of each sample. The 60 samples were equally divided into three groups, with 20 samples in each group, corresponding to prescribed target weights of 10 g, 15 g, and 20 g, respectively. In each group, 10 samples were used for the proposed method, and the remaining 10 samples were used for the Classic Sequential Algorithm. The cutting performance of each optimized scheme was compared through physical cutting rather than only computational simulation. An industrial fresh slicer with adjustable cutting angle and cutting interval (Fresh Slicer XQJ2-215, Hiwell Machinery Co., Ltd., Jinan, China) was used for the actual cutting experiments. After cutting, slice cross-sectional area, slice weight error, and residual material weight were measured for comparison between the two methods. The results were expressed as mean values with 95% confidence intervals. Differences in R1 and R2 between the proposed method and the Classic Sequential Algorithm were evaluated using independent-samples t-tests, with *p* < 0.05 considered statistically significant.

## 3. Results

### 3.1. Validation of the Weight–Length Distribution Model

#### 3.1.1. Validation of Volume Calculation

**Figure 5:**
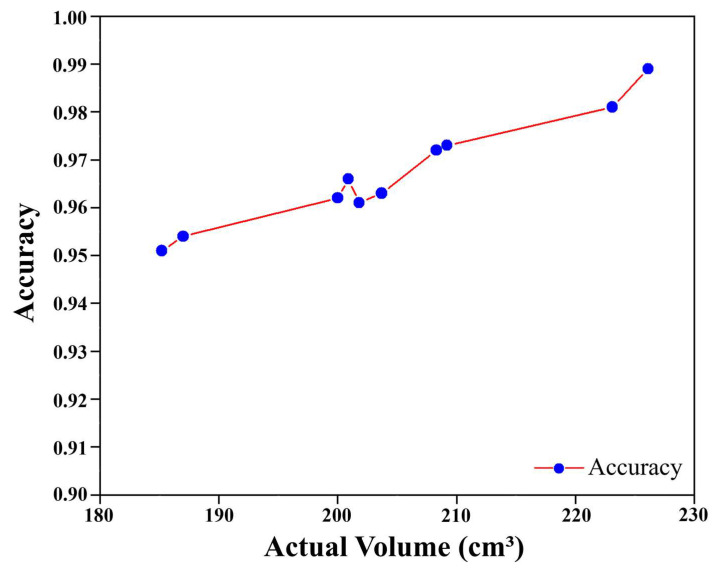
Variation in volume calculation accuracy across samples.

Ten fish fillet samples were selected for experimental validation. The measured weights and volumes of the experimental samples, as well as the predicted volumes obtained using the method described in Section 2.5.1, are presented in Table 1. The results showed that the volume calculation accuracy of the ten samples ranged from 95% to 99%, indicating a high overall accuracy. Further analysis revealed that the values calculated by the proposed method were slightly lower than the measured values. Figure 5 shows the overall variation trend of the calculation accuracy. The calculation accuracy of all samples exceeded 0.95, with the highest value of 0.989 observed for Sample 1 and the lowest value of 0.951 observed for Sample 9. The average accuracy was 0.967. Moreover, for samples with greater mass and volume, the calculated results were closer to the measured values. These results indicate that the proposed method achieved sufficient accuracy in volume calculation. According to the measured weight and volume data in Table 1, the average density of the fish fillets was calculated to be 1.084 g/cm^3^. The slight underestimation may be attributed to point-cloud smoothing and the planar approximation of the lower fillet surface during closed-volume reconstruction. Since the calculation accuracy of all samples remained above 95%, the reconstructed geometry was considered reliable for subsequent weight–length modeling.

#### 3.1.2. Validation of the Distribution Model

To quantitatively evaluate the accuracy of the weight–length distribution model, 10 samples were cut at equal intervals of 10 mm. The volume and weight of each sample were then obtained according to the proposed method, and the model-calculated values were compared with the measured values. As shown in Table 2, the weight prediction accuracy for all samples ranged from 94.4% to 96.6%, with an average accuracy exceeding 95%, indicating that the model achieved high predictive accuracy. Additional statistical analysis showed good agreement between the measured and model-calculated weights, with R^2^, RMSE, and MAE values of 0.994, 9.29 g, and 9.22 g, respectively. The mean prediction accuracy was 95.77%, with a 95% confidence interval of 95.30–96.24%. The mean relative error was 4.18%, and the residuals ranged from −12.01 g to −7.78 g, indicating a slight systematic underestimation by the model. This residual trend was consistent with the regional underestimation observed in the abdominal region.

**Figure 6:**
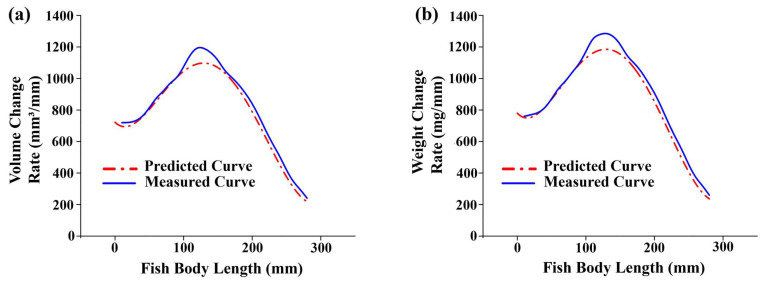
Comparison of predicted and measured distribution curves for a representative sample: (**a**) volume–length curve; (**b**) weight–length curve.

**Figure 7:**
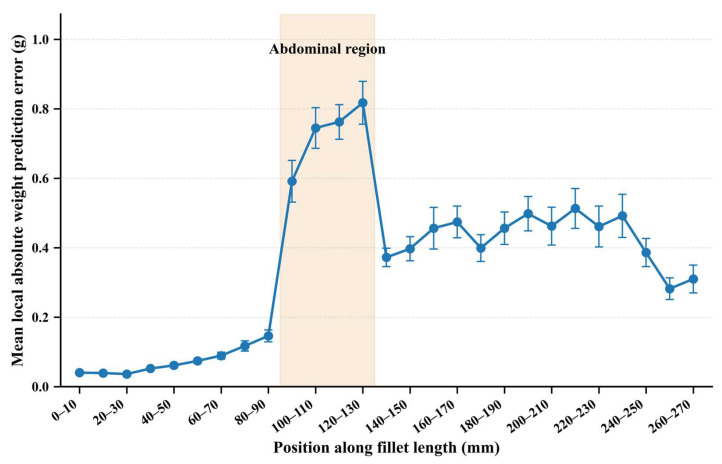
Local absolute weight prediction error distribution along the fillet length. Error bars indicate standard deviations, and the shaded region indicates the abdominal region.

Figure 6a,b present, the measured and modeled volume–length distribution curves and the measured and modeled weight–length distribution curves for a representative fish sample. Combined analysis of the distribution curves in Figure 6 indicates that the model accuracy exhibited distinct regional characteristics. In the anterior and posterior portions of the fillet, the predicted curves agreed closely with the measured curves, suggesting that three-dimensional reconstruction in these regions was relatively accurate and that the resulting volume and weight estimations were reliable. By contrast, in the abdominal region, the model showed a systematic underestimation tendency. As shown in Figure 7, the local prediction error increased mainly within the 90–130 mm interval, corresponding to the abdominal region of the fillets. This regional underestimation may be attributed to two factors. First, the abdominal region exhibits pronounced curvature variation. In the preliminary multi-radius reconstruction tests, ball radii of 5, 10, and 15 mm were compared using representative fillet point clouds. A radius of 5 mm retained more local details but produced obvious holes in sparse point-cloud regions, whereas a radius of 15 mm reduced holes but caused over-smoothing and loss of local geometric features. The 10 mm radius provided a better balance between surface continuity, local feature preservation, and reconstruction efficiency, and was therefore used for all samples. Second, differences in moisture, fat, and other tissue components between the middle region and other regions may increase the density non-uniformity of this region, thereby aggravating the discrepancy between the model-calculated weights based on the average-density assumption and the actual weights. Nevertheless, the overall weight prediction accuracy remained within 94.4–96.6%, indicating that the average-density approximation was acceptable under the current experimental conditions. For applications requiring higher regional prediction accuracy, local density correction or data-driven compensation may be considered when sufficient regional density or error-calibration data are available.

### 3.2. Validation Experiments for Multi-Objective Optimized Cutting

To evaluate the optimization performance of the proposed method under different prescribed target weights, the 60 fish fillet samples were equally divided into three groups according to the method described in Section 2.5.2, with 20 samples in each group. The three groups were used for cutting experiments at prescribed target weights of 10 g, 15 g, and 20 g, respectively. In each group, 10 samples were cut using the proposed method, and the remaining 10 samples were cut using the Classic Sequential Algorithm. Table 3, Table 4 and Table 5 present the comparative statistical results of the cutting experiments performed using the two methods. The evaluated indicators included the theoretical number of slices $\left(n^{\prime }\right)$, the actual number of slices $\left(n\right)$, the maximum weight deviation $\left({max}_{w}\right)$, the minimum weight deviation $\left({min}_{w}\right)$, the maximum relative weight error $\left(\delta_{max}\right)$, the mean relative error of weight $\left(MRE\right)$, and the relative standard deviation of slice area $\left(R_{1}\right)$. Since the residual material weight varies randomly under different prescribed target weights, $R_{2}$ was introduced to directly evaluate the optimization effect of the proposed method on residual material weight. Here, $R_{2}$ denotes the ratio of the actual residual material weight to the theoretical residual material weight. A value of $R_{2}$ less than 1 indicates an optimization effect, and a smaller value represents a greater degree of optimization.

**Figure 8:**
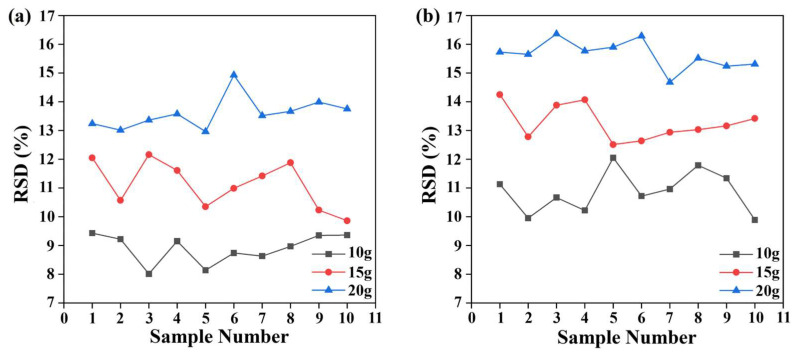
Line plots of the relative standard deviation of slice area (R_1_) under different prescribed target weights: (**a**) proposed method; (**b**) conventional sequential cutting method.

Figure 8 and Table 6 summarize the variation in the relative standard deviation of slice cross-sectional area (R1) under different prescribed target weights. The proposed method consistently produced lower R1 values than the conventional sequential cutting method under all three target weights, indicating that the proposed method improved the consistency of slice cross-sectional area. This improvement can be attributed to the joint optimization of cutting interval and cutting angle, which enables the cutting scheme to better adapt to the irregular geometry of fish fillets. In contrast, the conventional sequential cutting method mainly focuses on cumulative weight control and has limited ability to regulate slice shape consistency. The independent-samples t-test further showed that the reductions in R1 were statistically significant at target weights of 10 g, 15 g, and 20 g, with *p* values all below 0.001.

Table 7 summarizes the residual material ratio (R2) under different prescribed target weights. Compared with the conventional sequential cutting method, the proposed method reduced R2 by 56.4%, 48.3%, and 42.0% at target weights of 10 g, 15 g, and 20 g, respectively, with an average reduction of 48.9%. This indicates that the proposed method improved raw material utilization by reducing the proportion of residual material. In practical processing, residual material is not necessarily discarded. If it meets hygiene and product-specification requirements, it can be collected and used for minced fish, surimi-based products, restructured fish products, dried products, or other cooked products. Together with the reduced relative standard deviation of slice cross-sectional area, the results show that the proposed method can improve slice specification consistency while reducing material loss. The 95% confidence intervals further showed that the proposed method produced lower R1 and R2 values than the Classic Sequential Algorithm under all three target weights, and the between-method differences were statistically significant (*p* < 0.05).

**Figure 9:**
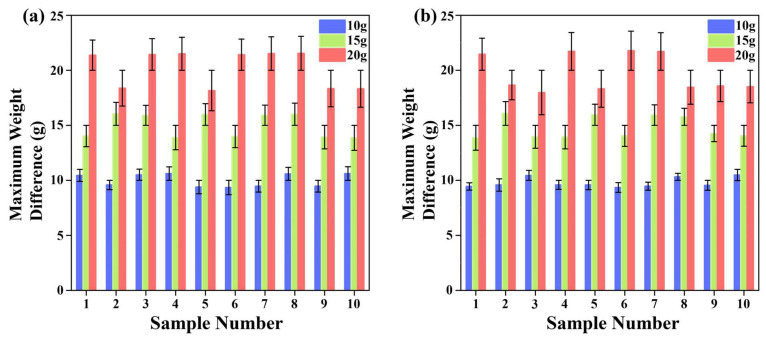
Weight error plots: (**a**) proposed method; (**b**) conventional sequential cutting method.

**Figure 10:**
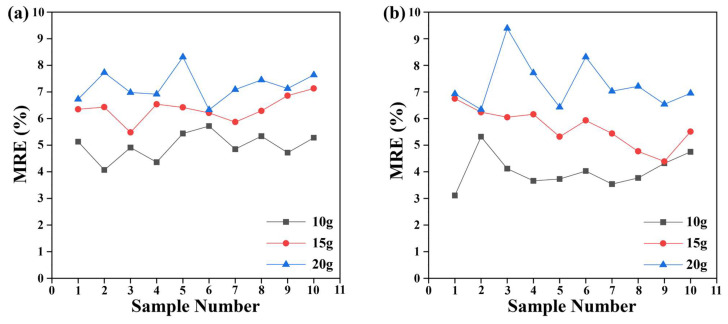
Line plots of mean relative error (MRE): (**a**) proposed method; (**b**) conventional sequential cutting method.

To further examine weight-control stability, Figure 9 shows the weight-error distributions obtained by the two methods. As shown in the figure, the error dispersion increased with the target slice weight. This trend is first related to the 5% relative weight constraint. When the target weights were 10 g, 15 g, and 20 g, the allowable absolute errors were 0.5 g, 0.75 g, and 1.0 g, respectively; thus, the fluctuation range of weight error increased with the target value. In addition, as discussed in Section 3.1.2, the predicted values of the weight–length distribution function were systematically lower in the middle region of the fillet, and the abdominal tissue density was non-uniform. These factors may further increase the dispersion of actual cutting weight errors. Figure 10 presents the mean relative error (MRE) of weight for the two methods. The results show that the MRE values of both methods decreased as the prescribed target weight decreased, indicating that cutting accuracy improved with decreasing prescribed target weight. Under the same prescribed target weight, the proposed method generally showed smaller fluctuations in MRE and higher cutting accuracy. When the prescribed target weight was relatively large, the MRE values of the proposed method were overall lower than those of the conventional sequential cutting method, demonstrating a clear advantage in slice weight accuracy. As the prescribed target weight decreased, the results of the conventional sequential cutting method gradually became better than those of the proposed method; however, the difference in accuracy between the two methods was not substantial. Overall, in the cutting experiments with different prescribed target weights, the MRE values of the proposed method were all below 8%, indicating higher overall accuracy and better suitability for practical production requirements.

**Figure 11:**
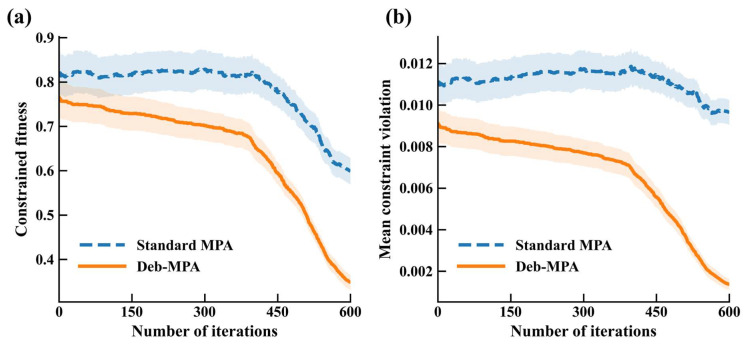
Convergence comparison between Standard MPA and Deb-MPA: (**a**) constrained fitness; (**b**) mean constraint violation. The curves represent the mean values of repeated runs, and the shaded regions indicate the 95% confidence intervals.

In addition to the cutting-validation results, the convergence behavior of Standard MPA and Deb-MPA was compared under the same population size and iteration number. As shown in Figure 11a, Deb-MPA obtained lower constrained fitness values than Standard MPA during the iterative process. The mean constrained fitness decreased from 0.60 to 0.35, corresponding to a reduction of 41.77%. Figure 11b shows that Deb-MPA also produced a lower mean constraint violation. The feasible-solution rate increased from 0.00% to 40.33%, and the mean constraint violation decreased from 9.65 × 10^−3^ to 1.38 × 10^−3^, corresponding to a reduction of 85.71%. These results indicate that Deb’s feasibility rules improved the ability of MPA to search feasible cutting schemes under weight, residual-material, and geometric constraints. The average computational time of a single Deb-MPA optimization run was 0.40 ± 0.12 s per fillet under the current parameter setting. The ten repeated runs were used to evaluate the stability of the stochastic optimization process, whereas a single run can generate one cutting scheme for practical implementation.

## 4. Discussion

From the perspective of measurement, line-laser scanning was used in this study to acquire three-dimensional contour information of fish fillets, providing continuous cross-sectional geometric data for subsequent weight-distribution modeling. Existing machine-vision methods can be used for fish weight estimation, size measurement, or cutting-position prediction; however, two-dimensional images mainly describe planar features, such as length, width, and area, and are insufficient for representing thickness variation and abdominal bulging in fish fillets [13,17]. In contrast, line-laser scanning is more suitable for describing volume variation along the fillet length and can therefore provide a data basis for establishing the weight–length distribution function [14]. In this study, the weight prediction accuracy ranged from 94.4% to 96.6%, and the mean relative weight errors in the cutting validation were all below 8%. These results indicate that the proposed measurement and modeling method can meet the requirements of cutting validation for the tested fish species, source, and chilled storage condition. It should be noted that this accuracy does not imply that the model parameters can be directly generalized to all fish species and processing conditions. When applied to different fish species, raw-material sources, fishing seasons, or temperature conditions, density measurement, weight–length distribution model fitting, and cutting-parameter validation are still required.

In the proposed fish-fillet cutting model, the starting and ending positions of each cut were defined as decision variables. These variables can be directly mapped to the tool feed distance and inclination angle, giving the optimization results a clear physical correspondence with actuator control parameters. Compared with the Classic Sequential Algorithm, which mainly controls slice weight, the proposed multi-objective method can actively coordinate slice shape consistency and raw-material utilization, providing a quantitative evaluation approach for variable-interval and variable-angle cutting schemes. For irregular fillets that are thick in the middle region and narrow and thin toward the tail, maintaining slice cross-sectional area consistency is important for processing quality but difficult to achieve. Meanwhile, reducing residual material is economically important in large-scale processing. Therefore, the allowable slice-weight deviation (±5%) was treated as a constraint in this study, allowing a reasonable trade-off in weight accuracy to improve shape consistency and raw-material utilization. The experimental results showed that the mean RSD of slice cross-sectional area was reduced by 13.06–18.13% compared with the sequential algorithm, and the actual residual material weight was reduced to 0.581–0.710 times the theoretical value. These results verify the improvement of the multi-objective method in overall cutting performance. Liu et al. [39] used slice weight and shape as dual objectives in squid slicing, indicating that the definition of optimization objectives depends on the specific processing scenario and that the model structure should match the actual process requirements. In this study, the dual-objective model explicitly expresses the priority of the three indicators through weighting coefficients. After weighted transformation, the model can be efficiently solved using a single-objective optimization algorithm, avoiding the computational cost and decision uncertainty associated with post-processing of Pareto solution sets.

For the proposed fish-fillet cutting model, the optimization algorithm needs to continuously search for feasible cutting positions within the fillet length range to obtain an optimized combination of multiple cutting positions. For this type of problem, population-based search mechanisms, such as MPA and PSO, are more suitable than single-point search methods, such as SA, for exploring the solution space and achieving efficient optimization. In Liu et al. [39], the multi-objective squid slicing model was solved using SA with 4000 iterations. In contrast, the Deb-MPA used in this study converged under a population size of 10 and 600 iterations, with an average optimization time of 0.40 ± 0.12 s per fillet. Deb-MPA inherits the advantages of standard MPA, including fewer control parameters and no need for penalty functions, and introduces Deb’s feasibility rules for constraint-guided search, thereby avoiding penalty-factor tuning and improving convergence stability. In comparison, PSO relies on global-best guidance and may be prone to premature convergence in objective functions with multiple local extrema; GA requires encoding and decoding of continuous variables, which may reduce iterative efficiency; and although NSGA-II can output a Pareto solution set, its computational pattern is not well matched with the weighted single-objective strategy used in this study [35]. The quantitative comparison in Table 8 shows that, under the same objective function, constraints, population size, and maximum iteration number, the mean effective convergence iteration of Deb-MPA was 185 ± 22, which was lower than those of PSO (338 ± 35) and GA (412 ± 35). This indicates that Deb-MPA could obtain a stable feasible cutting scheme more rapidly. In terms of the average optimization time per fillet, Deb-MPA required 0.40 ± 0.12 s, which was comparable to PSO (0.43 ± 0.11 s) and lower than GA (0.55 ± 0.15 s). This suggests that Deb-MPA achieved a better balance between convergence efficiency and computational cost. In addition, Deb-MPA mainly requires setting the population size and maximum iteration number, with relatively low parameter sensitivity [34,35]. This makes it more convenient for local engineering deployment when fish species or processing conditions change.

The proposed method still has several limitations for broader application. The average-density approximation was used for volume-to-weight conversion, while fillet density and deformation may vary with species, raw-material source, storage condition, fishing season, and temperature. Therefore, when the method is applied to fresh, chilled, or other temperature-conditioned samples, density measurement, weight–length model fitting, and cutting-parameter validation should be performed under the corresponding processing conditions. From an industrial perspective, the workflow has the potential for continuous operation if scanning, reconstruction, optimization, and cutter response are synchronized, although practical throughput will still depend on scanning resolution, point-cloud processing speed, optimization time, conveyor stability, and cutter response speed. Future work will focus on integrating machine vision, AI-assisted optimization, servo or robotic cutting systems, and adaptive learning to improve online correction, parameter search, variable-angle execution, and model updating.

## 5. Conclusions

This study proposed a multi-objective optimization cutting method for irregularly shaped fish fillets based on line-laser scanning, weight–length distribution modeling, and constrained optimization. Based on fillet shape measurement data acquired by line-laser scanning, a mathematical model describing the relationship between fillet weight and length was established. In addition, a quantitative description method for cutting quality and economic performance was developed by taking slice weight, slice cross-sectional area, and residual material weight as decision parameters. By transforming the control variables, namely cutting thickness and cutting angle, into decision variables representing the starting and ending displacements of each cut, a multi-objective optimization model for fish fillet cutting was established. To address the limitation of the standard Marine Predators Algorithm (MPA) in handling constrained optimization problems, Deb’s feasibility rules were introduced to improve the algorithm and enable optimal determination of cutting positions. The results showed that the prediction accuracy of slice weight reached 94.4–96.6%, indicating that the developed weight–length function could accurately characterize the weight distribution of fish fillets. Compared with the conventional sequential cutting method used for target-weight straight cutting, the proposed method produced mean RSD values of slice cross-sectional area of 8.9%, 11.112%, and 13.601% at target weights of 10 g, 15 g, and 20 g, respectively. The corresponding residual material weights were reduced to 0.581, 0.693, and 0.710 times the theoretical residual material weights. Moreover, the optimized cutting scheme effectively improved slice shape consistency and raw material utilization, and the mean relative errors of slice weight obtained by the proposed method were all below 8%, satisfying practical production requirements. These results demonstrate the superiority and practical applicability of the proposed multi-objective optimization cutting method in the precise processing of irregularly shaped fish fillets and provide effective methodological support for the intelligent and standardized cutting of high-value fish products.

## Figures and Tables

**Table 1:** Comparison between measured and calculated volume data of fish fillet samples.

Sample No.	Length (mm)	Measured Volume (cm^3^)	Weight (g)	Density (g/cm^3^)	Calculated Volume (cm^3^)	Accuracy
1	285	226.1	248.4	1.099	223.842	0.989
2	280	223.1	241.3	1.082	218.641	0.981
3	253	201.8	218.5	1.083	193.728	0.961
4	255	203.7	220.9	1.084	195.552	0.963
5	260	209.2	226.3	1.082	202.924	0.973
6	259	208.3	225.7	1.084	202.051	0.972
7	247	200	216.3	1.082	192.331	0.962
8	248	200.9	217.7	1.084	192.864	0.966
9	232	185.2	200.2	1.081	175.943	0.951
10	235	187.0	202.6	1.083	177.652	0.954

**Table 2:** Comparison between measured and model-calculated values of the distribution model.

Sample No.	Length (mm)	Measured Volume (cm^3^)	Measured Weight (g)	Model-Calculated Volume (cm^3^)	Model-Calculated Weight (g)	Position of Maximum Error (mm)	Accuracy
1	285	226.1	248.4	221.371	239.0807	120~130	0.962
2	280	223.1	241.3	215.941	233.2163	120~130	0.966
3	253	201.8	218.5	192.612	208.021	100~110	0.952
4	255	203.7	220.9	196.201	211.8971	100~110	0.959
5	260	209.2	226.3	202.336	218.5229	110~120	0.965
6	259	208.3	225.7	201.012	217.093	100~110	0.961
7	247	200	216.3	191.729	207.0673	100~110	0.957
8	248	200.9	217.7	190.457	205.6936	100~110	0.944
9	232	185.2	200.2	177.113	191.282	90~100	0.955
10	235	187.0	202.6	179.502	193.8622	90~100	0.956

**Table 3:** Cutting results for the prescribed target weight of 10 g.

Method	Sample No.	Sample Weight (g)	*w_a_* (g)	*n*′	*n*	max*_w_* (g)	min*_w_* (g)	*δ*_max_ (%)	*MRE* (%)	*R*_1_	*R*_2_
Proposed Method	1	236.5	10	23	23	0.55	0	5.5	5.13	9.43	0.54
	2	223.3	10	22	22	0.42	0	4.2	4.07	9.22	0.73
	3	249.8	10	24	24	0.51	0	5.1	4.91	8.01	0.73
	4	213.3	10	21	21	0.61	0.2	6.1	4.36	9.15	0.68
	5	235.3	10	23	23	0.61	0.1	6.1	5.44	8.14	0.52
	6	208.8	10	20	20	0.65	0.1	6.5	5.72	8.74	0.57
	7	217.1	10	21	21	0.53	0.2	5.3	4.85	8.63	0.41
	8	207.9	10	20	20	0.59	0.1	5.9	5.34	8.97	0.37
	9	225.4	10	22	22	0.53	0	5.3	4.72	9.35	0.63
	10	225.2	10	22	22	0.62	0.2	6.2	5.28	9.36	0.58
Conventional Sequential Cutting Method	1	237.7	10	23	23	0.33	0	3.3	3.11	11.13	1.63
	2	219.3	10	21	21	0.56	0	5.6	5.32	9.95	1.63
	3	244.1	10	24	24	0.45	0.1	4.5	4.12	10.67	0.82
	4	213.7	10	21	21	0.41	0	4.1	3.66	10.22	0.60
	5	235.4	10	23	23	0.42	0.2	4.2	3.73	12.05	1.38
	6	206.8	10	20	20	0.44	0	4.4	4.03	10.72	0.68
	7	215.1	10	21	21	0.37	0	3.7	3.54	10.96	2.40
	8	196.2	10	19	19	0.32	0.1	4.2	3.47	11.79	0.94
	9	229.2	10	22	22	0.45	0	4.5	4.32	11.34	1.26
	10	223.6	10	22	22	0.51	0	5.1	4.75	9.89	1.98

**Table 4:** Cutting results for the prescribed target weight of 15 g.

Method	Sample No.	Sample Weight (g)	w_a_ (g)	n′	n	max_w_ (g)	min_w_ (g)	δ_max_ (%)	*MRE* (%)	R_1_	R_2_
Proposed Method	1	212.2	15	14	14	0.97	0.2	6.5	6.35	12.05	1.57
	2	249.6	15	16	16	1.04	0.1	6.9	6.43	10.57	0.40
	3	229.6	15	15	15	0.91	0.2	6.1	5.48	12.16	0.76
	4	223	15	14	14	1.11	0.1	7.4	6.54	11.61	0.77
	5	237.9	15	15	15	0.99	0	6.6	6.42	10.35	0.60
	6	202.3	15	13	13	1.01	0.1	6.7	6.21	10.99	0.44
	7	228.3	15	15	15	0.92	0.3	6.1	5.87	11.42	0.80
	8	217.5	15	14	14	1.01	0.1	6.7	6.29	11.88	0.61
	9	194.9	15	12	12	1.14	0	7.6	7.13	9.86	0.55
	10	207.4	15	13	13	1.07	0.2	7.1	6.86	10.23	0.43
Conventional Sequential Cutting Method	1	208.9	15	13	13	1.13	0.2	7.5	6.75	14.25	1.65
	2	243.2	15	16	16	1.08	0.2	7.2	6.24	12.78	1.83
	3	232.4	15	15	15	1.04	0.2	6.9	6.05	13.88	1.67
	4	220.5	15	14	14	1.07	0.2	7.1	6.16	14.07	1.72
	5	233.9	15	15	15	0.96	0.1	6.4	5.32	12.51	0.92
	6	201.3	15	13	13	0.96	0	6.4	5.93	12.64	1.40
	7	230.5	15	15	15	0.93	0	6.2	5.44	12.94	1.12
	8	215.2	15	14	14	0.77	0	5.1	4.77	13.03	0.96
	9	193.8	15	12	12	0.95	0.1	6.3	5.51	13.42	1.10
	10	204.1	15	13	13	0.74	0	4.9	4.39	13.16	1.02

**Table 5:** Cutting results for the prescribed target weight of 20 g.

Method	Sample No.	Sample Weight (g)	*w_a_* (g)	*n*′	*n*	max_*w*_ (g)	min_*w*_ (g)	*δ*_max_ (%)	*MRE* (%)	*R*_1_	*R*_2_
Proposed Method	1	231.8	20	11	11	1.38	0.2	6.9	6.73	13.24	0.79
	2	197.7	20	9	9	1.62	0.2	8.1	7.73	13.01	0.61
	3	247.6	20	12	12	1.44	0.3	7.2	6.98	13.36	0.62
	4	209.3	20	10	10	1.5	0.2	7.5	6.92	13.58	0.72
	5	218.6	20	10	10	1.84	0.3	9.2	8.31	12.96	0.48
	6	243.7	20	12	12	1.42	0	7.1	6.33	14.93	1.45
	7	207.7	20	10	10	1.52	0.2	7.6	7.09	13.52	0.64
	8	248.2	20	12	12	1.54	0.2	7.7	7.45	13.67	0.53
	9	213.2	20	10	10	1.66	0.1	8.3	7.13	13.99	0.59
	10	211.9	20	10	10	1.68	0.2	8.4	7.64	13.75	0.63
Conventional Sequential Cutting Method	1	231.9	20	11	11	1.46	0.1	7.3	6.93	15.73	1.35
	2	199.7	20	9	9	1.34	0	6.7	6.34	15.65	0.99
	3	237.1	20	11	11	2.02	0.2	10.1	9.39	16.36	1.40
	4	212.3	20	10	10	1.72	0.1	8.6	7.72	15.77	0.80
	5	226.6	20	11	11	1.68	0.1	8.4	6.43	15.9	0.90
	6	233.1	20	11	11	1.78	0	8.9	8.31	16.29	1.35
	7	203.6	20	10	10	1.71	0.1	9.5	7.03	14.68	1.94
	8	247.6	20	12	12	1.54	0.2	7.7	7.21	15.52	1.35
	9	219.5	20	10	10	1.42	0.2	7.1	6.54	15.24	1.03
	10	216.2	20	10	10	1.48	0	7.4	6.95	15.31	1.10

**Table 6:** Comparison of mean R_1_ values under three prescribed target weights.

Method		Target Slice Weight (g)	
	10	15	20
Conventional Sequential Cutting Method (%)	10.872	13.268	15.645
Proposed Method (%)	8.9000	11.112	13.601

**Table 7:** Comparison of mean R_2_ values under three prescribed target weights.

Method	Target Slice Weight (g)		
	10	15	20
Conventional Sequential Cutting Method	1.332	1.341	1.223
Proposed Method	0.581	0.693	0.710

**Table 8:** Quantitative comparison of Deb-MPA, PSO, and GA under identical optimization conditions.

Algorithm	Population Size	Maximum Iterations	Repeated Runs	Mean Effective Convergence Iteration	Average Optimization Time per Fillet (s)
Deb-MPA	10	600	10	185 ± 22	0.40 ± 0.12
PSO	10	600	10	338 ± 35	0.43 ± 0.11
GA	10	600	10	412 ± 35	0.55 ± 0.15

## Data Availability

The original contributions presented in this study are included in the article. Further inquiries can be directed to the corresponding author.
